# Domestic Summary

**Published:** 1840-02-22

**Authors:** 


### DOMESTIC SUMMARY.

### CLINICAL MEDICINE.

The following resolutions are published at the request of the class attending the lectures on clinical medicine, delivered during the winter of 1839–'40, at the Philadelphia Hospital:

"A portion of the medical class of the University of Pennsylvania, who have attended the lectures at the Philadelphia Hospital, being desirous of expressing their sense of the value and importance of clinical instruction, and of their obligations to Dr. W. W. GERHARD for the able course of lectures delivered by him at that institution during the present session, have met together for that purpose. Therefore,

*Resolved,* 1. That we consider clinical instruction the most important method of teaching the pathological states of the system, and of familiarizing the mind of the student with the means of correcting the aberrations from the standard of health.

2. That we consider the course of lectures on clinical medicine, now being delivered at the Philadelphia Hospital, of great value, particularly because of the truly scientific, as well as practical manner, in which diseases, involved in much obscurity, are elucidated.

3. That we consider Dr. Gerhard eminently qualified to give instruction in clinical medicine and pathological anatomy, and that we particularly admire his unequalled skill in illustrating the diseases of the thoracic organs.

4. That a committee of ten be appointed to present a copy of these resolutions to Dr. Gerhard, and to tender him our thanks for the zeal and ability which he has manifested in the interests of the class.

5. That these resolutions be signed by the chairman and secretary, and that a copy of them be presented to the Faculty through their Dean.

6. That a copy of these resolutions be also sent to the medical journals and newspapers of this city, with a request that they publish them.

The committee appointed under the fourth resolution, consists of the following gentlemen:—Drs. C. Quarles and R. Kownselar, and Messrs. T. B. Lamar, M. A. Page, T. R. Spencer, W. H. Van Buren, Allen Gunn, H. Selden, T. L. Walker, and J. R. Justice. On motion, the chairman was added to the committee.

J. A. PLEASANTS, M. D.,
*Chairman.*

L. S. JOYNES, M. D., *Secretary.*"

---

From an interesting paper by Dr. Detmold, published in the New York Journal of Medicine and Surgery, we extract the following interesting remarks relative to the treatment of club-foot. A lecture on the subject, by Dr. Mütter, of this city, with wood cuts of the apparatus necessary, was published in the Examiner of last year.

The extended application of this operation renders it of great interest to surgeons:

"Generally in children less than a year old, the muscular contraction will yield to the mere extension with proper instruments, although, of course, the age alone decides nothing. We have operated upon children of three months of age; and we have cured, without dividing a tendon, congenital club-feet in patients of four years of age. Besides, it makes a difference whether mechanical means have been already used; for, although they may not have been efficient enough to cure the disease, still they may have operated as a check against contraction. In cases not congenital, the causes and the duration of the disease are important in deciding the question, whether to operate or not. Recent cases after paralysis, and before the deformity has reached a high degree, seldom require the knife; we have cured a lad of fifteen years of age without operation, where the deformity in both feet had existed thirteen years, but where various kinds of shoes had been used during most of the time, which, although the patient only walked the worse for them, still had kept a sort of control over the contraction."

"The operation itself is very simple. After the patient is placed in the most suitable and convenient position, an assistant, or the surgeon himself, with his left hand extends the heel to make the tendon more tense, so that it shall not yield before the edge of the knife. The principles to be observed in performing the operation are, to cut, not by drawing the knife, but by *pressing it against the fibres*,—in this way there is no danger of wounding other tissues,—to divide the tendon where it is most prominent under the skin, to separate it completely, not leaving any of its fibres undivided which might afterwards be torn by the extension; to pass the knife as closely round the tendon as possible, so as to make the internal wound not larger than is necessary, and to make the wound in the integuments as small as possible.

A good deal has been said about the shape of the knife, whether it should be straight, concave, or convex. We formerly used a concave knife, but now use, in preference, a straight one; the shape of the knife is immaterial, provided the blade be narrow. The one we are now in the habit of using, is in its broadest portion not quite one-twelfth of an inch. In dividing round tendons, and tendons or muscles which are so placed that it is easy to pass the knife underneath without fear of wounding other parts, it is advisable to use a straight or slightly concave knife, to introduce it under the tendon with the edge upward, and cut from within outward; whereas, in cutting flat muscles, aponeuroses, or ligaments, we prefer a convex knife, which we introduce under the skin, and divide the tendon by pressing the knife upon the contracted and tense fibres, cutting from without, inward. Some surgeons, in dividing the tendo Achillis, make two external wounds, that is to say, they suffer the point of the instrument to penetrate the skin on the opposite side of the tendon: others pretend to see a great advantage in making only a single external wound. We have operated both ways, and found no difference in them. When we used the concave knife, we generally made two wounds; but now, with the straight knife, we make only one; but these wounds are so small that if a surgeon should think the operation easier and safer by bringing the point of the instrument out on the other side of the tendon, the second wound would be no objection against this method; on the contrary, we would certainly advise him to adopt it. In fact, provided the general principles already laid down are followed, the mere mechanical execution of the operation is so simple that every surgeon of moderate skill will be able to perform it.

While the instrument is passing through the fibres of the tendon, we always hear a peculiar grating sound, and, when the division is completed, a distinct jerk, from the retraction of the severed extremities, is heard and felt. Sometimes, however, the surrounding cellular tissue is so tense, and the muscle so rigid in its contraction, that the extremities of the cut tendon cannot separate so suddenly, and consequently the jerk is not perceived. We should therefore make it a rule after withdrawing the knife, always to ascertain with the finger whether the tendon has been completely divided, and if we feel some fibres remaining tense under the skin, we should introduce the point of the knife again, through the same external wound, and divide these fibres. This accident may happen if the instrument is passed too closely round the tendon, so that the point enters between the fibres, those beneath the back of the knife of course remaining undivided. We have never met with any untoward accident. Once, only, in dividing the tendo Achillis in a little girl of nine years of age, we had a slight hæmorrhage; and although the blood came out, at first, in a jet, it stopped almost immediately, the child losing hardly half an ounce. Scoutetten relates two cases of hæmorrhage after the division of the tendo Achillis; in one case the little patient lost from eight to nine ounces of blood: he succeeded, in both cases, in stopping the bleeding by compression. Any accident that may occur has, of course, to be treated according to the general rules of surgery.

The external wounds are so small that they require no other dressing than a small piece of court-plaster: for precaution's sake, we put a small compress wet with cold water, and a loose bandage over it, and in every case in which we have operated, the wounds have healed by the first intention. Immediately after the division of the tendo Achillis, the patient generally feels a numbness in the limb for a short time, and after that an occasional twitching in the muscles of the calf, but the pain ceases immediately after withdrawing the knife.

We formerly used to apply a bandage similar to that employed after rupture of the tendon, for the purpose of bringing the cut extremities together; but we have found even that unnecessary, because the ankle-joint is generally so stiff that the extremities cannot separate far enough to make it probable that they should not unite again. In purely spasmodic cases, however, where the contraction is very strong, and the ankle-joint flexible, so as to admit, by the descending of the heel, a greater separation of the extremities of the tendon, we still adhere to our former practice.

We now arrive at a very important question, viz., whether to begin extension immediately after the division of the tendon, so as to make the cut extremities at once separate as far as possible, or whether to allow the tendon time to unite again, and then to commence stretching gradually the yet soft intermediate substance; and if the latter method be preferred, how long will it be advisable to wait before the process of reunion shall be far enough advanced to exclude the fear of the divided extremities remaining separated, or of causing inflammation?

Some of the French orthopedists have adopted and advocated the method of immediate extension; if our readers have had the patience thus far to follow our reasoning and views on the whole subject of tenotomy, they will readily perceive that we must be opposed to this proceeding; and we feel confident that the majority, nay, in fact, every prudent practitioner, will agree with us, especially after having impartially considered the advantages and disadvantages of either method.

All the advantages which the advocates of immediate extension claim for their method, are the saving of time and of pain; but if we compare the result of their practice with that of others, for instance, Stromeyer's, Little's, and our own, we do not find it in their favour. We have, besides, repeatedly, after operating upon both feet in the same patient, applied the extending apparatus to one foot immediately, and to the other several days after the division, and have experienced no difference between the two feet; or, if any, the immediate extension was the more painful. In one case which we had operated upon, we were obliged, by circumstances beyond our control, to defer the application of the apparatus for three weeks after the operation, during which time the foot was left entirely to itself; and although the deformity was great, we succeeded just as well as in other cases where we had applied the apparatus much sooner. This proves the advantages of the method, which by its advocates is called an improvement of Stromeyer's, to be, at least, very problematical; and, indeed, this might be expected, *a priori*, for even if we allow two or three days to the tendon, for the exudation of the reuniting plastic lymph, this does not gain, in so short a time, consistency and firmness enough to offer any considerable resistance to extension. That resistance which we experience in deferring the extension a few days after the division, results mainly from the contraction of the tarsal ligaments, and is, of course, the same as in immediate extension. The advantages of this method become still more doubtful if the theory be correct, that tenotomy acts dynamically by relieving the spasm of the divided muscle; for, according to this view, it would hardly make any difference at what period after the operation extension is commenced; and such we find to be the fact in purely spasmodic talipes.

The disadvantages and perils of immediate extension are, on the other hand, almost self-evident, so much so that even if a series of successful cases, where no dangerous symptoms followed that procedure, were adduced in favour of the method, we should be inclined to consider them rather as so many lucky escapes, than as proofs of the safety of the method.

The dangers which we fear from this, so termed, improvement upon Stromeyer's method, and why we are opposed to it, are of a two-fold character; first, the tendon may not unite again, and thus the patient may be maimed for life. This is no vain apprehension; for it has been proved by experiment on animals, that a tendon will unite again if the extremities separate two inches from each other; but that reunion seldom takes place if the distance is greater, and never if it amounts to four inches. Although the rigidity of the tarsal ligaments in talipes seldom admits of such a wide separation, yet in recent cases, where the ligaments are not so much affected, it may occur, especially as the application of the extending apparatus by the pressure, (which in fastening it to the leg is unavoidable,) may, at the same time, act as a stimulus to convulsive contraction of the gastrocnemii.

The other danger is, that inflammation, with all its consequences, as suppuration and sloughing of the tendon, may be caused by forcibly stretching the fresh wound, a danger which will also be increased by the impediment to free circulation in the limb, arising from the compression of its blood-vessels by the fastening of the apparatus.

It certainly does not require much arguing to prove that these two results may follow immediate extension; and if they do, that they are so injurious in their consequences, that even if the yet doubtful advantages of the method, the saving of time and pain, could be positively proved, (which we by no means concede,) we doubt much whether prudence could sanction it.

The period when the extension may be commenced without incurring the danger of either causing inflammation or preventing reunion of the tendon, cannot be positively fixed; the circumstances which must guide us to determine this point, are the rigidity of the contracted gastrocnemii muscles and of the tarsal ligaments, together with the progress of the healing process. The former must be ascertained before the operation, the latter by an examination of the external wound. If we find that the space between the separated extremities of the tendon is filled up with plastic lymph, which is ascertained by a slight unelastic swelling under the skin, without pain or redness, we may safely commence extension without fear of disturbing the reunion. This exudation of plastic lymph generally takes place within twenty-four hours after the operation; but in cases where the ankle-joint is very flexible, and where the extremities of the tendon have separated more widely than usual, it may be prudent to wait a day or two longer.

After having, by the division of the tendon, overcome the greatest obstacle to the reduction of the deformity, this must be effected mainly by mechanical means; for the operation itself produces hardly any immediate change in the appearance of the limb; of the value of external applications, frictions, and champooing, we have said a few words already; they are useful assistants in giving tone to the muscles; but for the alteration of the shape of the foot, the mechanical means can alone be relied upon. Various machines have been invented and used to accomplish this object; in fact, almost every surgeon who has paid some attention to this branch of surgery, has contrived an instrument of his own which he thinks preferable to all others, because he is accustomed to use it. This only proves that the construction of the machine is of little importance, provided it fulfils the indications of the case, which are,

1st, To bring down the heel.

2d, To bring down the inner margin of the foot; and,

3d, To relieve the contraction of the inner margin of the foot."

"From time to time, the machine has to be taken off; especially if the patient complains of soreness of the skin, the foot must be examined and washed with a spirituous lotion. A circumstance which makes the skin more subject to become blistered and sore, besides the unavoidable pressure, is the profuse perspiration in which the foot is often kept during the whole treatment; it is sometimes so profuse that the mere bandaging up of the foot does not appear sufficient to us to account for it. We believe that the peculiar coldness of the skin in talipes, arises from the vessels of the skin participating in the spasmodic affection of the muscles; and that when the spasm in the muscles is relieved, the spasm of the skin is also relieved by sympathy; and hence the profuse perspiration during the treatment. We are the more inclined to account for it in this manner, as we have observed the same symptom after the operation, where no machine had yet been applied.

If we find the epidermis raised to a blister, which occurs easiest on the instep, the blister must be opened; and if the cutis beneath is not much inflamed, it is sufficient to cover the part with simple cerate, when we may put the machine on again. Generally the skin heals under the same pressure which produced the blister; but if the cutis has been more seriously affected by the pressure, and exhibits a suppurating surface, the application which we have found most effective is a solution of nitrate of silver with tincture of opium, which we apply with a compress, and generally put the machine on again, even in this case. Sometimes we find a sore of a torpid character, which remains in the same state for a long while, neither getting worse, nor showing any inclination to heal, in that case, an ointment of red precipitate of mercury is the best application. If the patient loses his appetite, has a furred tongue, complains of headach, and is feverish, the machine must be taken off, and the foot examined. In this case, we generally find either an abscess forming, which requires opening, or a small spot of skin that is becoming gangrenous; under these circumstances, the use of the machine must be interrupted, unless we can arrange the straps so as to favour the part affected.

The leaving off the machine causes, of course, a delay in the cure, yet this is not so much as most writers on the subject seem to apprehend. We have occasionally been obliged to leave the machine off for several weeks; but, after it had been applied again for a day or two, we found that we not only had lost no ground, but that we were progressing again very fairly. We even think it sometimes advisable to let the foot alone for a short while; we have treated feet that had been doing very well for a length of time, but where at once every new attempt at extension became painful and ineffective; we have then been led to suppose that perhaps the tarsal ligaments, or the periosteum of the tarsal bones, might be in an irritated condition, perhaps a little swelled,—an effect that is not unlikely to be occasionally produced by the treatment. With this impression, we have taken the machine off, put a loose bandage round the foot, and left it to itself, in a quiet, horizontal position, for about a week; and wherever we have done so, under such circumstances, we have been able to proceed with the extension, after a week's intermission, much faster than before. Besides, it is sometimes a relief to the patient, and advances the cure much, to change the machine, for a while, and apply Scarpa's shoe."

"The time which is requisite to reduce the deformity, so that the patient can wear a shoe and begin to walk, depends upon so many different circumstances, as the degree of the deformity, the age and constitution of the patient, and many other things besides, that it is almost impossible to fix it with any degree of precision. However, we may consider from one to three months as a fair average, although we have treated feet that have withstood our orthopedic efforts much longer, sometimes eight months and more; and, even after the deformity is reduced, it generally requires a great deal of care and attention, and a long-continued use of Scarpa's shoe, or a similar contrivance, especially in old cases of varus, before the patient is able to enjoy the full benefit of the operation. In fact, many patients with varus, after they have reached a certain age, require some kind of mechanical support for years, and, if they are left without it, the foot turns in again. In cases where both feet are deformed, the deformity is generally worse than where only one foot is affected, probably because the primary cause has acted more powerfully, and, consequently, affected both feet. In such cases, also, one foot is always much worse than the other, and often takes double the time before it gets straight. Besides, we have observed that those cases where the foot does not yield much before the operation, where the joints are rather stiff, are the most favourable, in this respect, that they have less inclination to turn back after they are once straightened; whereas, vari that can easily be straightened to a certain degree, on account of the joints being loose, require a longer time before these loose joints gain strength in their new position.

We mention this particularly, because the profession may be misled by reports of very speedy and perfect cures. For instance, Dr. Mütter, of Philadelphia, performed his first operation of the kind in the latter part of June, 1838, and early in 1839 he published a lecture on the subject. According to the annexed resumé, Dr. M. had operated during that time upon twenty-eight cases; of these, there were sixteen patients between the sixth and thirtieth year, and one between the thirtieth and fiftieth year. At the time of the publication of his lecture, Dr. M. reports twenty of these cases *perfectly cured;* one was cured within from ten days to four weeks; nine from four weeks to two months, and ten from two to four months. This report is very favourable; yet the term *perfectly cured*, ought to be well understood, for we doubt much whether all these twenty *perfectly cured* patients could walk within four months (the longest period mentioned) with any degree of ease, and without artificial support of some kind or other; and that is what we expect ourselves, and especially what our patients expect from us, before they consider themselves *perfectly cured.** We wish it to be distinctly understood that, in making this remark, we do not mean to express the least doubt in Dr. M.'s success, or in the correctness of his report. We merely wish to guard the profession, as well as the public, against wrong impressions. Suppose, for instance, a surgeon operates upon his first case of the kind; every thing is doing as well as can be, but, although the foot may be almost straight, still the patient cannot walk, even at the expiration of *the four months*, and, if he can, only poorly, and not without some artificial support; will not the surgeon, who perhaps has, as yet, no experience on the subject, but who has read such favourable reports, I say, will not the surgeon, in his modesty, provided he has any, think that he has perhaps failed in some point or other, and, still more, will not the patient be inclined to believe it? Dr. Mütter, for whose talents we entertain the highest regard, must forgive us that we have taken his report as an illustration of this point; we might have taken many others besides his, for writers on the subject seem inclined to fall into the same fault. If we can succeed in cases of long standing, success, independent of the time it may require, is so creditable to the art, (we do not mean to the individual artist, the surgeon,) but to *the art* of surgery, that all embellishment is superfluous and unnecessary.

* Only a few days ago, our fee, for an operation of this kind, was refused to us by the father of the child, although the child had been running about with a perfectly straight foot for more than six months, but we had advised the family to keep the shoe on for a while yet. The father, as we say, refused to pay our fee, because he did not consider his child cured as long as "that 'ere tackle" had to be worn.

Cases of grown patients that admit of a perfect cure (as we understand the term) in so short a time, are rarely met with, although we have seen them; but a great number of our patients whom we have operated upon long ago, and in whom we have every reason to be satisfied with the success of the operation, we see still improving daily. In children it requires, of course, much less time; we have seen children walk perfectly well a week after the operation; yet even then, we find it necessary to support the ankle-joint for a considerable length of time. In children where the deformity is not very great, we generally find Scarpa's shoe alone sufficient to effect a cure, either with or without an operation, as the case may require. In very young infants, we at first only apply a bandage, until they are a month or two old, and then use the shoe.

We have met with cases of varus that had been cured for some time, and the patients had been doing well, when a new contraction took place. Here the patients had either been neglected, the shoes having been left off too soon, or they had suffered from another disease which probably caused this new contraction, or perhaps the original cause of the deformity was not yet quite removed,—in short, a relapse took place. We believe we have mentioned already that Little and Stromeyer ascribe this to a contraction of the intermediate substance of the tendon, but that we are disposed to regard it as a new contraction of the muscles, and the success of our treatment bears us out in our opinion. In these cases we do not hesitate to divide the tendo Achillis the second time; and after this, we have rarely found it necessary to use any extending apparatus, the mere division of the tendon being generally sufficient to relieve this new spasm."

